# The HIV glycan shield as a target for broadly neutralizing antibodies

**DOI:** 10.1111/febs.13530

**Published:** 2015-10-23

**Authors:** Katie J. Doores

**Affiliations:** ^1^ Department of Infectious DiseasesFaculty of Life Sciences and MedicineKing's College LondonGuy's HospitalUK

**Keywords:** broadly neutralizing antibody, envelope trimer, glycosylation, HIV, vaccine

## Abstract

The HIV envelope glycoprotein (Env) is the sole target for HIV broadly neutralizing antibodies (bnAbs). HIV Env is one of the most heavily glycosylated proteins known, with approximately half of its mass consisting of host‐derived N‐linked glycans. The high density of glycans creates a shield that impedes antibody recognition but, critically, some of the most potent and broadly active bnAbs have evolved to recognize epitopes formed by these glycans. Although the virus hijacks the host protein synthesis and glycosylation machinery to generate glycosylated HIV Env, studies have shown that HIV Env glycosylation diverges from that typically observed on host‐derived glycoproteins. In particular, the high density of glycans leads to a nonself motif of underprocessed oligomannose‐type glycans that forms the target of some of the most broad and potent HIV bnAbs. This review discusses the changing perception of the HIV glycan shield, and summarizes the protein‐directed and cell‐directed factors controlling HIV Env glycosylation that impact on HIV bnAb recognition and HIV vaccine design strategies.

**Abbreviations**
bnAb: broadly neutralizing antibody
EM: electron microscopy
Env: envelope glycoprotein
ER: endoplasmic reticulum
PNGS: potential N‐linked glycosylation site
UPLC: ultraperformance liquid chromatography

## Introduction

**Figure 1:**
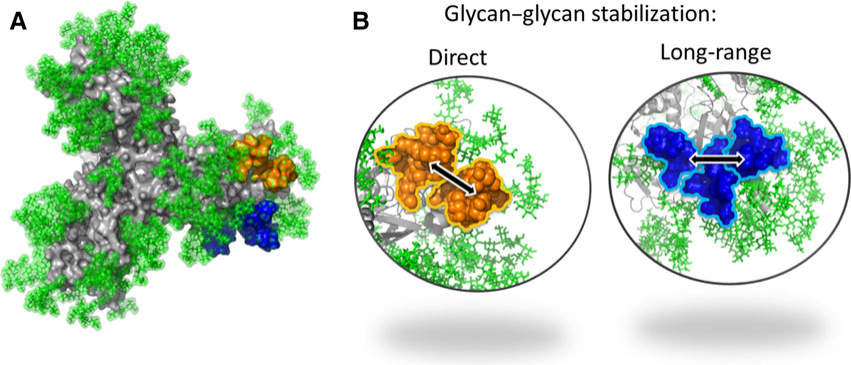
The mannose patch consists of microclusters of glycans. (A) Model of the glycosylated HIV Env trimer 40 based on the recent structures of BG505 SOSIP.664 31, 58, 59 viewed from the trimer apex. The N‐linked glycans are shown in green, and the protein in grey. Glycans are modelled as high‐mannose. (B) Glycan modelling showing examples of direct glycan–glycan stabilization between N386 and N392 (shown in orange), and long‐range glycan–glycan stabilization between N488 and N332 mediated through interaction with glycan N295 (shown in blue).

The HIV envelope glycoprotein (Env) is one of the most heavily glycosylated proteins known, with ~ 50% of its mass consisting of host‐derived N‐linked glycans, and it is the sole target for HIV broadly neutralizing antibodies (bnAbs) (Fig. 1A). Env glycans are important for assisting correct protein folding, for viral infectivity 1, and for modulating the interaction with the host immune system 2, 3. Until fairly recently, the glycans coating the surface of HIV Env were considered to form a ‘glycan shield’ that hid conserved protein regions of HIV Env from the adaptive immune system, and thus impeded recognition by potential neutralizing antibodies 3, 4. However, it is becoming increasingly apparent that these glycan structures can also act as targets for HIV bnAbs, with many of the most potent and broadly active HIV bnAbs contacting HIV Env glycans 5.

## The HIV glycan shield as a target for bnAbs

The elicitation of HIV bnAbs will likely be a key step for the development of a successful HIV vaccine. Approximately 10–30% of HIV‐infected individuals elicit bnAbs after 2–3 years of infection 6. These bnAbs, when passively administered to macaques at low serum concentrations, are able to protect from infection in SHIV challenge models 7, 8, suggesting that, if they could be elicited through vaccination, they would be effective in reducing HIV transmission rates. In order to design vaccines that might re‐elicit such bnAbs, it is important to characterize their interaction with HIV Env at the molecular level 9, 10. To date, a large number of HIV bnAbs have been isolated and characterized, revealing regions on HIV Env that are vulnerable to bnAbs. These regions include the CD4‐binding site (e.g. b12, VRC01, and PGV04 11, 12, 13), the membrane proximal external region on gp41 (e.g. 4E10 and 10E8 14, 15), and proteoglycan epitopes centred at three Env regions 16, 17, 18. Until relatively recently, only one bnAb, 2G12, had been identified that binds the HIV glycan shield 19, 20. 2G12 has an unusual and extremely rare domain‐exchanged structure, whereby the heavy chains cross over to form a multivalent binding surface allowing binding to multiple N‐linked glycans in one region with high avidity 21, 22. 2G12 has been shown to solely interact with N‐linked glycans on gp120, including N295, N332, N339, and N392 23. However, a large proportion of the HIV bnAbs isolated over the last 5 years have also been shown to bind to the HIV glycan shield, with three main epitopes having been identified: the N332 glycan site and V3 loop (e.g. PGT121, 10‐1074, PGT128, and PGT135 17, 24, 25, 26, 27); the N160 glycan site and V1/V2 loops (e.g. PG9, PGT145, and CH04 17, 18, 28); and the N‐linked glycans near the gp41–gp120 interface (e.g. PGT151 and 35O22 16, 29). Unlike 2G12, these antibodies have a conventional non‐domain‐exchanged, Y‐shaped structure 25, 26, 30. The Fab regions contact both the HIV Env glycans and protein components, and have much higher breadth and potency than 2G12 (for example, PGT128 neutralizes 72% of viruses with a median IC_50_ of 0.02 μg·mL^−1^, as compared with 2G12, which neutralizes 32% of viruses with a median IC_50_ of 2.38 μg·mL^−1^) 17, 26, 30, 31, 32. Typically, these bnAbs are highly mutated, and use long CDRH3 regions to penetrate through the glycan shield to contact the protein regions beneath as well as the glycan structures 17, 26, 30, 31, 32. For example, PGT128 has been shown to bind N‐linked glycans at positions N332 and N301, in addition to making backbone‐mediated protein contacts with the V3 loop 17, 30. Interestingly, several of the N332‐binding bnAbs have been shown to bind promiscuously, and can use different arrangements of N‐linked glycans for neutralization 24, 33. These factors enable these bnAbs to neutralize a diverse range of viral isolates and to limit the ability of the virus to escape through shifting or removing N‐linked glycan sites 33. HIV glycosylation is therefore emerging as a likely vulnerability that can be targeted in vaccine design.

As HIV Env is so heavily glycosylated, and many of the HIV bnAbs target glycan binding and/or glycan‐dependent epitopes, it is critical to characterize the glycan structures present on native HIV Env to design Env‐based immunogens that correctly mimic the glycosylation of circulating virus. Although some characterization of the HIV glycan shield was carried out when HIV was first discovered 34, 35, 36, 37, 38, the isolation of glycan‐binding HIV bnAbs has led to a renewed effort and the use of more advanced analytical techniques to precisely characterize the glycan structures present on HIV Env. Here, I discuss the changing perception of the HIV glycan shield between 2009 and 2015, and the factors controlling HIV Env glycosylation that make the HIV glycan shield an attractive target, but also a challenge, for vaccine design.

## HIV Env structure

HIV Env facilitates host cell entry through interaction with the CD4 receptor and CCR5 or CXCR4 coreceptors on target cells. HIV Env is first expressed as a gp160 precursor, before proteolytic cleavage by furin 10 in the trans‐Golgi into heterodimers of a surface‐exposed glycoprotein, gp120, and a transmembrane glycoprotein, gp41. The HIV Env trimer consists of three of these gp120–gp41 heterodimers, which are noncovalently associated. Depending on the HIV strain, gp120 has a median of 25 potential N‐linked glycosylation sites (PNGSs) 39, with some strains having over 30 PNGSs. Gp41 is less heavily glycosylated, having, on average, four PNGSs. Some of these PNGSs are highly conserved across clades, whereas others are less well conserved, being situated within the variable regions of gp120 40.

Upon entry into target CD4 cells, HIV must replicate and produce new viral particles to sustain and spread infection. HIV therefore hijacks the host cell protein synthesis and glycosylation machinery to produce the proteins and glycoproteins required for virus production. As such, HIV Env is entirely glycosylated by the host cell glycosylation machinery, and follows a very strict ordered assembly. Glycosylation begins in the endoplasmic reticulum (ER) with the addition of Glc_3_Man_9_GlcNAc_2_ to Asn residues in the consensus sequence Asn‐X‐Ser/Thr (where X can be any amino acid except Pro). Action of the glucosidase I and II enzymes then removes the first two terminal glucose residues to give GlcMan_9_GlcNAc_2_. The monoglucosylated gp160 interacts with calnexin and calreticulin chaperones, allowing gp160 to fold through interactions with other ER chaperones and disulfide isomerases, such as ERp‐57. Inhibition of this folding process through the use of glycosidase inhibitors such as *N*‐butyldeoxynojirimycin significantly reduces viral infectivity 1. After removal of the final glucose residue by glucosidase II, the terminal mannose residue of the D2 arm is trimmed by the ER α‐mannosidase I enzyme to give Man_8_GlcNAc_2_ before the protein moves to the cis‐Golgi, where the glycans are trimmed further by the Golgi α‐mannosidase I enzymes to Man_5_GlcNAc_2_. The protein then moves to the medial Golgi where GlcNAc transferase I adds a β1,2‐linked GlcNAc, opening up the pathway to further trimming by Golgi α‐mannosidase II, and elaboration and diversification of glycan structures by glycosyltransferase enzymes to generate complex‐type and hybrid‐type glycans. The type and level of glycosyltransferase enzymes present vary between cell types, and the structures of complex‐type and hybrid‐type glycans present on a glycoprotein are therefore strongly dependent on producer cell type.

## The HIV glycan shield

**Figure 2:**
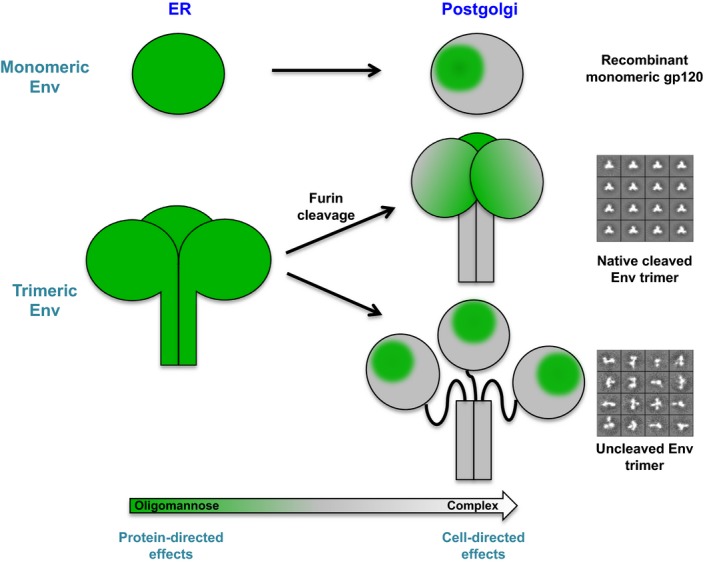
Both protein‐directed and cell‐directed effects determine the glycosylation of HIV Env. The gp160 precursor is glycosylated in the ER at glycosylation sequons NXT/S (where X can be any amino acid except proline) with oligomannose‐type glycans. As gp160 moves through the Golgi compartments, the glycoprotein is exposed to glycan‐processing enzymes, which convert oligomannose‐type glycans (green) into complex‐type glycans (grey). The dense clustering of glycans on the gp120 outer domain limits the accessibility of the ER mannosidase I enzyme, leading to a patch of unprocessed oligomannose‐type glycans (referred to as the mannose patch), which is present on all forms of HIV Env, including recombinant monomeric gp120. The quaternary and compact structure of native trimeric HIV Env, as revealed by the EM images, further restricts the accessibility of processing enzymes, resulting in the secreted protein having a predominant oligomannose population. Uncleaved HIV Env trimer has a more open structure, as observed on the EM images. This allows easier access of the glycan‐processing enzymes, and a larger population of the oligomannose glycans are therefore processed to complex‐type glycans. The glycans on gp41 are not restricted in the same way as on gp120, and complex‐type glycan structures therefore predominate. This figure is adapted from 45.

As the N‐linked glycan structure added to the NXT/S motif is not template‐driven in the same way as protein synthesis, mammalian cell‐derived glycoproteins typically consist of many different glycoforms, in which the same protein backbone is glycosylated with a number of different glycan structures. Analysis of HIV Env glycosylation was first conducted over 25 years ago by the use of HPLC analysis of glycans released from gp120 shed from HIV‐infected lymphoblastoid H9 cells 34, 38. Two main subpopulations of glycans were identified on gp120: underprocessed oligomannose glycans, and processed complex‐type glycans. RP‐HPLC analysis of glycans released from recombinant gp120 expressed in CHO cells also identified these two subpopulations 35, 36, 37. These initial studies, along with a number of more recent studies utilizing mass spectrometry and ultraperformance liquid chromatography (UPLC) 40, 41, 42, 43, 44, 45, show that, although HIV Env is entirely processed by the host cell glycosylation machinery, there is divergence from the default pathway of ‘self’‐glycosylation that generates a window for potential immune recognition. Therefore, the overall glycosylation profile of native HIV Env is determined by both protein‐directed and cell‐directed effects, and will be discussed below (Fig. 2).

## Protein‐directed features of HIV Env glycosylation

The protein‐directed effects controlling HIV Env glycosylation arise from the density and position of N‐linked glycans present on gp120 (encoded by the primary protein sequence), as well as the three‐dimensional shape of the trimeric glycoprotein. These effects are largely independent of producer cell type.

### The mannose patch

The extremely high density of glycans on HIV Env is unusual among enveloped viruses, and this is reflected in the differences in glycan structures present on different viruses 5. For example, unlike recombinant attachment glycoproteins from Nipah, Hendra and Machupo viruses (the last of these being the causative agent of Bolivian haemorrhagic fever), which have highly processed complex‐type glycan structures 46, 47, HIV has an unusual dominant population of underprocessed oligomannose‐type glycans (Fig. 2). The mechanism by which these oligomannose‐type glycans arise differs from that observed for other enveloped viruses; for example, hepatitis C virus buds from the ER, thus bypassing the Golgi compartments, and displays only Man_8–9_GlcNAc_2_ glycans 48, and Uukuniemi virus buds from the medial Golgi 49. Site‐specific analysis of glycans on recombinant monomeric gp120 35, 50, 51, 52, X‐ray structural analysis of gp120 53 and molecular‐level characterization of bnAb epitopes 26, 30, 31 all show the majority of oligomannose‐type glycans to cluster on the outer domain of gp120 (Fig. 1A). A study by Doores *et al*. 42 has shown that the dense clustering of glycans on HIV Env prevent the ER α‐mannosidase I enzyme from accessing its Man_9_GlcNAc_2_ substrate, resulting in a population of glycans that are trapped as Man_8–9_GlcNAc_2_. More recently, Pritchard *et al*. 40 have modelled the interaction of the N332 glycan on the high‐resolution BG505 SOSIP.664 trimer structure with ER α‐mannosidase I, and shown that the high density of glycans around N332 generates significant steric constraints that impede access to the terminal D2 mannose residue. Combined, these studies demonstrate that this area of underprocessed high‐mannose glycans is an inherent property of the HIV Env glycoprotein, and not an effect of incomplete cellular processing or an unusual secretory pathway. This region is often referred to as the ‘mannose patch’, and is a distinguishing feature of HIV Env glycosylation 5.

Analysis of a number of HIV Env expression systems, including recombinant gp120 monomers, recombinant trimers, and virus preparations, shows that this population of oligomannose glycans is a conserved feature of HIV Env that is independent of producer cell type 34, 41, 45, 50, 52, 54, 55, 56. This divergence from host cell glycosylation provides a window for discrimination between host and virus that is exploited by some HIV bnAbs 5, 26, 30. This region is highly accessible to bnAbs, and has recently been described as a supersite of vulnerability, owing to the diverse range of bnAbs that recognize this region of the trimer 26, in particular N332‐binding HIV bnAbs.

### Importance of trimerization and cleavage

Because of their use in HIV vaccine clinical trials and ease of preparation, the majority of HIV Env glycosylation studies have been conducted on recombinant, monomeric gp120 expressed in CHO or HEK 293T cells. More recently, attention has been focused on characterization of glycosylation on native forms of HIV Env, in particular HIV Env derived from virus particles 41, 42, 44, recombinant soluble trimers 45, and membrane‐tethered trimers 43, 44, 45. These studies have given additional insights into how the protein‐directed features of trimerization and gp120–gp41 cleavage can impact on HIV Env glycosylation.

Studies of HIV Env isolated from both replication‐competent virus grown in human CD4^+^ T cells and pseudovirus grown in HEK 293T cells have shown that the glycosylation of native HIV Env is not as heterogeneous as might be expected 41, 42, 44. Not only is the proportion of oligomannose glycans high (62–79% across different strains 41), but the types of complex sugars present are fairly restricted, consisting of mostly sialylated bi‐antennary, tri‐antennary and tetra‐antennary complex‐type glycans 44. This increase in oligomannose‐type glycan level as compared with monomeric gp120 (23–50% across different strains) is also observed for recombinant trimers and membrane‐tethered trimers 43, 45. The production of recombinant soluble trimers has been hampered by both instability and aggregation, and vaccine efforts have therefore been focused on designing stabilized HIV Env trimers that might better mimic native HIV Env found on viral particles. Particular success has been achieved with the BG505 SOSIP.664 trimer through introduction of a disulfide bond to covalently link the gp41 and gp120 subunits, and the addition of trimer‐stabilizing mutations between gp41 subunits 57. This protein has been shown to antigenically mimic native HIV Env through selective binding to a panel of HIV bnAbs but not non‐neutralizing HIV Abs 57, and has been used to generate the first high‐resolution HIV Env trimer structures 31, 58, 59. BG505 SOSIP.664 forms compact, homogeneous trimers as detected by negative stain electron microscopy (EM) (Fig. 2) 57, and analysis of the glycosylation present on the gp120 subunit showed a dominant oligomannose population (60–71%), similar to the level observed on native HIV Env from virions 44, 45. The glycosylation on gp41 did not display this oligomannose feature, and will be discussed further below.

The increased level of underprocessed oligomannose‐type glycans on native HIV Env likely arises from additional protein–glycan and glycan–glycan interactions that occur in the context of the trimer. This is supported by recent structural data for the BG505 SOSIP.664 recombinant trimer, showing the close proximity of many of the N‐linked glycan sites at the trimer interfaces, and the extension of the oligomannose‐type glycans beyond just the gp120 outer domain 31, 59. A recent structure of HIV Env in complex with bnAb 35O22 showed that the HIV glycan shield extends from gp120 into gp41, with gp120 glycans N88, N234 and N241 being adjacent to gp41 glycan N625 10, 29, 59. When the N160 and N332 sites of vulnerability are viewed in the context of the HIV Env structure, using previous crystal structures of glycan‐dependent bnAbs, it is clear that these sites are larger than previously thought and, in fact, overlap to some extent 31. Interestingly, when Man_9_GlcNAc_2_ glycans were modelled on each sequon of the HIV Env trimer structure, only 19% of the protein surface was shown to be solvent‐accessible and 3% to be accessible to the Fab domain of a full antibody 59.

The impact of trimerization on HIV Env glycosylation is also evident from studies by the Crispin laboratory with uncleaved recombinant trimers (Fig. 2). To achieve full cleavage of the BG505 SOSIP.664 gp160 precursor, this protein must be coexpressed with the protease furin 45, 57. However, endogenous furin present in HEK 293T cells is sufficient to give a population of both cleaved and uncleaved trimers. Analysis of the glycosylation of uncleaved BG505 SOSIP.664 shows a much higher degree of glycan processing than in the cleaved form 45. Negative stain EM of the uncleaved form shows a heterogeneous configuration, whereby the gp120 subunits are splayed from the gp41 core, and only remain tethered because of their uncleaved nature (Fig. 2) 60. This aberrant configuration likely allows better enzyme accessibility to glycan structures at the trimerization interfaces, and thus accounts for the greater degree of glycan processing. These data suggest that correct folding of trimeric HIV Env can be rescued through cleavage, and that cleavage destines HIV Env for correct folding and correct glycan processing. Indeed, negative stain EM images of other uncleaved gp140 trimer immunogens (e.g. CZA97.012, UG37, and CN54, which all contain foldon trimerization domains) also point to this aberrant trimeric structure, and they all have a greater degree of glycan processing 45. Although no direct glycan profiling was conducted, AlSalmi *et al*. 61 observed that cleavage impacts on the mobility of HIV Env on SDS/PAGE gel, with an uncleaved gp140 migrating more slowly than the cleaved variant. Combined, these studies of native HIV Env suggest that the processing of HIV Env glycans is further restricted by the trimerization of monomeric subunits, whereby additional protein–glycan and glycan–glycan interactions between protomers further limit the accessibility of glycan‐processing enzymes.

### Stability in the face of sequence diversity

HIV Env is under constant pressure by the host immune system in an HIV‐infected individual. Addition and deletion of glycan sites is a common response to antibody‐mediated selection pressure; therefore, a concern regarding the targeting of HIV Env glycans for vaccine design is the shifting nature of the HIV glycan shield and subsequent loss of bnAb glycan epitopes 4. A recent study by Pritchard *et al*. 40 attempted to address these concerns by investigating the integrity and robustness of the mannose patch in the face of sequence variation. PNGSs were systematically removed from the HIV strain BaL by the use of site‐directed mutagenesis, and the impact on the glycosylation profile of recombinant gp120 determined with UPLC. In general, removal of individual glycan sites had a minimal impact on glycan processing, suggesting that the density of glycans on gp120 is sufficiently high to tolerate individual glycan site deletions. However, larger than expected perturbations in the oligomannose isomers were observed upon loss of some glycosylation sites, e.g. N262, N386, and N392. As the N262A mutation often results in poorly infectious virus 62, 63, 64, and N262 is 100% conserved across strains 40, removal of N262 was thought to cause misfolding of gp120 and therefore disruption of the glycan clustering. However, structural modelling of N386 and N392 showed that these two glycans form a microcluster within the mannose patch, whereby removal of one partner leads to greater glycosidase trimming of the neighbouring sugar (Fig. 1B), and this is an example of direct glycan–glycan stabilization. Overall, the persistence of the oligomannose‐type glycans in the presence of glycan site deletion highlights the importance and robustness of the mannose patch for HIV vaccine design 5, 40. Indeed, Sok *et al*. 33 have shown that some N332‐dependent bnAbs can bind alternative arrangements of N‐linked glycans in the absence of N332.

Several studies have reported underglycosylation of founder/transmitted viruses as compared with chronic viruses, particularly for clades A and C, with founder/transmitted viruses generally having lower numbers of PNGSs 65, 66. Go *et al*. 54 characterized the glycosylation of two recombinantly expressed transmitted/founder Envs, and compared these to two Envs isolated from clade‐matched, but not patient‐matched, chronically HIV‐infected individuals. Although they identified differences in site occupancy and the amounts of complex‐type glycans present, this study was severely limited by the unrelated nature of the virus sequences studied. Wei *et al*. 4 first introduced the concept of the ‘evolving glycan shield’ as a mechanism of escape from autologous neutralizing antibodies in 2003. However, changes in overall HIV Env glycan composition during HIV infections has yet to be studied with longitudinal HIV Env sequences from an HIV‐infected individual, and it would be interesting to explore the impact that any changes in glycosylation might have on bnAb development and virus transmission.

## Cell‐directed features of HIV Env glycosylation

As mentioned above, the producer cell in which a virus is prepared or a protein is expressed can impact on the structure of complex‐type and hybrid‐type glycans present on HIV Env (Fig. 2). This is particularly evident for the glycosylation of gp41 and the type of sialic acid linkage of complex‐type glycans 44, 45.

### gp41 glycosylation

As mentioned above and in contrast to gp120, the glycosylation of gp41 consists of predominantly endo‐β‐*N*‐acetylglucosaminidase H (Endo H)‐resistant complex‐type structures, and indicates a lesser degree of protein‐directed processing 44, 45, 67, 68. This is likely attributable to the lower density of glycans on gp41 than on gp120, resulting in a higher degree of glycan processing. Comparison of glycans released from BG505 SOSIP.664 expressed in either CHO or HEK 293T cells shows that, although the glycosylation of gp120 is fairly similar, the glycan structures on gp41 are considerably different, highlighting the impact of the producer cell 45. Analysis of the glycans on gp41 released from pseudovirions produced in HEK 293T cells shows a predominant and heterogeneous population of mostly sialylated tri‐antennary and tetra‐antennary glycans that is consistent with the glycan specificity of PGT151 16, 44, 69. For some viral isolates, there appears to be a residual population of virions that are not sensitive to PGT151 neutralization, as shown by low neutralization plateaus 16, 70. It has been proposed that incomplete neutralization may arise because of the heterogeneity of gp41 glycosylation resulting in virus particles displaying glycan structures at critical contact sites (N611 and N637) that PGT151 cannot bind 16, 44, 69, 70, 71. This greater degree of heterogeneity may therefore highlight a limitation in targeting the glycans on gp41 for vaccine design.

### Nature of sialic acid linkage of complex‐type glycans

A recent study by Pritchard *et al*. 44 highlighted the producer cell type dependence of the nature of the sialic acid linkage of complex‐type glycans on native HIV Env. Selective neuraminidase digests showed that HIV Env from virions produced in human CD4^+^ T cells had sialylated complex‐type glycans that were predominantly α2,6‐linked, whereas HIV Env from pseudovirions produced in HEK 293T cells had sialylated complex‐type glycans that were entirely α2,3‐linked. Interestingly, activation of CD4^+^ T cells has been shown to lead to downregulation of ST6GAL1, the enzyme responsible for α2,6‐linked sialylation, and a subsequent decrease in sialylated glycans 72, 73. These data may suggest that HIV infection can lead to a remodelling of cellular N‐linked glycans by somehow reversing the ST6GAL1 downregulation.

Given the immunomodulatory roles of terminal sialic acid residues, in particular the interaction of α2,6‐linked sialic acid residues with CD22 on the surface of B cells, it is plausible that these differences in sialic acid linkage may have important implications for HIV immune responses and potential vaccine responses 74, 75. Furthermore, several studies examining the glycan specificity of HIV bnAbs using glycan microarrays have highlighted differences in specificities for α2,3‐linked and α2,6‐linked sialic acid residues. For example, PGT121 and PG16 preferentially bind α2,6‐sialylated biantennary glycans 25, 76, whereas PGT151 preferentially binds α2,3‐sialylated tetra‐antennary glycans 16. However, these bnAbs neutralize both HEK 293T‐derived pseudovirus and peripheral blood mononuclear cell‐derived virus with very similar IC_50_s, suggesting that this fine specificity is not important in the context of the full bnAb epitopes 16, 70.

## Microheterogeneity at individual PNGSs

As mentioned above, a number of studies have been carried out to determine the precise glycan structures present at each PNGS on gp120 and gp41 35, 50, 51, 52. These studies have used recombinantly expressed proteins, owing to the ease of preparation and the large amounts required for site‐specific glycan analysis. Site analysis data have recently been utilized to understand how microheterogeneity of HIV Env glycosylation can impact on HIV bnAb neutralization sensitivity 56. For some HIV strains, there is a minor population of virions that are resistant to neutralization by the N332‐dependent bnAb PGT135 26, 70. PGT135 contacts the glycans at N332, N392, and N386, and, although these sites are all situated within the mannose patch, site‐specific analysis of gp120_BaL_ shows that there are varying degrees of heterogeneity in oligomannose‐type glycans at these sites that impact on the ability of PGT135 to neutralize the virus 26, 40, 56. In particular, site analysis data and structural data 26 highlight that a Man_9_GlcNAc_2_ glycan at N392 appears to prevent PGT135 binding, owing to a steric clash between the terminal D2 arm mannose residue and the CDRH3 loop of PGT135. Sixteen per cent of the glycan at N392 is Man_9_GlcNAc_2_, which is consistent with the 80–85% plateau observed for BaL neutralization. These findings have important implications for vaccine design, as they suggest that a glycan bnAb response will likely require some degree of plasticity in the way in which it binds glycans to counteract the microheterogeneity described. PGT121, for example, is able to counteract this heterogeneity by contacting the conserved core glycan residues of N‐linked glycans, and is therefore able to recognize both complex‐type and oligomannose‐type glycans 25, 77.

Site‐specific analysis has also recently been used to determine the impact of glycan site deletions on the microheterogeneity of glycan structures present at N332 of gp120_BaL_, which is a major target for HIV bnAbs 40. Removal of glycans directly neighbouring N332 had limited impact on the types of glycan present, with only a redistribution of Man_9_GlcNAc_2_ to Man_8_GlcNAc_2_ glycans being observed. Interestingly, removal of glycan N448, which does not directly neighbour N332, also leads to a redistribution of Man_9_GlcNAc_2_ to Man_8_GlcNAc_2_ glycans, with the change likely being mediated through the N295 glycan with which it clusters. This is an example of long‐range glycan–glycan stabilization (Fig. 1B). These modest effects on N332 glycan structure are also reflected in the apparent ability of N332‐dependent high‐mannose‐binding bnAbs to still bind the PNGS‐deleted proteins 17, 33, 40. Overall, this suggests that the density of glycans surrounding N332 is sufficiently high to limit the impact on microheterogentiy at N332 when single neighbouring PNGSs are deleted.

The limitation of HIV Env glycosylation studies being carried out with recombinant proteins has been highlighted by the differences in glycosylation observed between native HIV Env and both recombinant monomeric gp120 and non‐native uncleaved trimers 40, 41, 42, 43, 44, 45. A key focus in the field now needs to be the site‐specific analysis of native HIV Env, either isolated from viral preparations, or by the use of antigenically native recombinant trimers such as the BG505 SOSIP.644 trimer. A recent study by Go *et al*. 43 has begun to address this by carrying out site‐specific analysis on membrane‐tethered HIV Env trimers produced in CHO cells. However, this study was limited by the use of uncleaved trimers, as outlined above. Site‐specific analysis of native HIV Env is vital to further characterize the epitopes of glycan‐binding HIV bnAbs, but also to aid vaccine design strategies aimed at re‐eliciting such bnAbs. What would be interesting to know is the level of heterogeneity within the complex‐type glycan population, and whether there are any protein‐directed effects that are controlling the types of complex‐type and hybrid‐type glycans at specific sites, in particular those targeted by HIV bnAbs, e.g. N137, N156/N160, and N611/N637 16, 18, 24, 78.

## O‐linked glycosylation

O‐linked glycosylation on HIV Env is less well studied than N‐linked glycosylation. Unlike for N‐linked glycosylation, there is no clearcut consensus sequence with which to distinguish an O‐glycosylated Ser or Thr from a nonglycosylated residue. Although most O‐linked glycoyslation occurs in Ser/Thr‐rich regions, isolated residues can also be glycosylated. Therefore, direct biochemical analysis is required to determine the O‐linked glycosylation on HIV Env. The majority of analyses have, again, been carried out with recombinant soluble gp120, and have shown the presence of mucin‐type O‐linked glycans 79, 80. A recent study by Stansell *et al*. 81 used site‐specific analysis to show that T499 on recombinant gp120 was almost fully glycosylated, with a disialylated core 1 mucin‐type O‐linked glycan being the most abundant. However, when the corresponding recombinant gp140 protein was analysed, < 50% of the sample was glycosylated, and the O‐glycans consisted of shorter chains than those observed on gp120. Furthermore, when virion‐associated HIV Env prepared in the human T‐cell line Sup‐T1 was analysed, no O‐linked glycosylation was observed at T449. These data suggest that the transmembrane component of HIV Env may limit the accessibility of transferase enzymes and prevent O‐linked glycosylation at this site 81. In contrast, two studies have identified O‐linked glycosylation on membrane‐anchored HIV Env trimers. First, a study by Go *et al*. 43 showed that a membrane‐tethered, uncleaved HIV Env trimer expressed in CHO cells was glycosylated at both T499 and T606. Second, a study by Yang *et al*. 82 showed that virion‐associated HIV Env prepared in the human T‐cell line ACH‐2 was also glycosylated at T499 with GalGlcNAc. Therefore, it is not yet clear whether protein‐directed and cell‐directed effects also control O‐linked glycosylation of HIV Env in a similar way to N*‐*linked glycosylation 40, 41, 42, 44, 45, or whether the O‐linked glycosylation observed is characteristic of misfolded proteins. Further research is needed in this area.

## Summary and future perspectives

Although, as described above, there is still further characterization of HIV Env glycosylation to be carried out, a clear picture of the factors controlling HIV Env glycosylation is now emerging (Fig. 2). The three‐dimensional structure of HIV Env and the dense clustering of PNGSs determine the divergence of HIV Env glycosylation from typical host cell glycosylation. This divergence creates nonself glycan motifs that are targeted by HIV bnAbs elicited during natural infection. The producer cell controls the remaining complex‐type and hybrid‐type glycans. Although the human immune system has overcome many of the challenges associated with eliciting antibodies against glycan antigens during HIV infection, it is now vital for vaccine researchers to overcome these challenges with immunogen design.

The research conducted over the last 5 years has provided a template of native HIV Env glycosylation for vaccine researchers to compare the glycosylation of potential HIV immunogens. Immunogens based on the full HIV Env trimer, e.g. BG505 SOSIP.644, have the greatest chance of matching the glycosylation of native virion‐associated HIV Env, as they can rely on the three‐dimensional protein structure to direct glycosylation. However, having the correct native HIV Env glycosylation profile is not sufficient for elicitation of HIV bnAbs. Immunization with either BG505 SOSIP.644 or virus‐like particles displaying native HIV Env trimers have elicited strong but only autologous tier 2 neutralizing antibodies 83, 84 (tier 2 viruses have moderate sensitivity to antibody‐mediated neutralization) 85. Initial mapping of the immune serum shows that neutralizing antibodies include glycan‐dependent epitopes, although further characterization is required 83, 84. These studies highlight the importance of using native‐like HIV Env trimers to elicit robust tier 2 neutralizing responses, but also highlight the need to mimic the processes occurring during natural infection, where it can take 2–3 years of chronic infection to broaden the neutralizing response. Therefore, having the correct HIV Env glycosylation profile is a consequence of having a correctly folded native HIV Env trimer, and is only one important factor in the search for an HIV vaccine.

Alternative vaccine strategies involve immunogens based on truncated forms of gp120 designed to elicit one particular neutralizing epitope. Here, the ability to match the glycosylation of native HIV Env will be much more difficult, as the protein structure will not control glycosylation in the same way as full‐length HIV Env. Although glycosidase inhibitors have been used to increase the abundance of either Man_9_GlcNAc_2_ glycans 86 or oligomannose‐type glycans, by the use of kifunensine or GlcNAc transferase I‐deficient cells, respectively, these strategies do not generate immunogens that mimic the microheterogeneity of glycosylation on HIV Env. As mentioned above, this is likely important for generating bnAbs that can tolerate the heterogeneity on virion‐associated HIV Env and neutralize all viral particles.

In summary, the HIV glycan shield is now considered to be an important target for HIV vaccine design. Both protein‐directed and cell‐directed effects determine HIV Env glycosylation, and the protein‐directed effects lead to a nonself motif of underprocessed oligomannose‐type glycans that forms the target of some of the most broad and potent HIV bnAbs. The biochemical and structural studies described above have encouraged the HIV vaccine field to consider the glycans present on potential immunogens in addition to the protein components.

## Author contribution

KJD wrote the manuscript.
